# Absence of AGG Interruptions Is a Risk Factor for Full Mutation Expansion Among Israeli FMR1 Premutation Carriers

**DOI:** 10.3389/fgene.2018.00606

**Published:** 2018-12-13

**Authors:** Noam Domniz, Liat Ries-Levavi, Yoram Cohen, Lilach Marom-Haham, Michal Berkenstadt, Elon Pras, Anne Glicksman, Nicole Tortora, Gary J. Latham, Andrew G. Hadd, Sarah L. Nolin, Shai E. Elizur

**Affiliations:** ^1^IVF Unit, Sheba Medical Center, Tel Hashomer, Israel; ^2^Sackler Faculty of Medicine, Tel Aviv University, Tel Aviv, Israel; ^3^Danek Genetic Institute, Sheba Medical Center, Tel Hashomer, Israel; ^4^New York State Institute for Basic Research in Developmental Disabilities, Staten Island, NY, United States; ^5^Asuragen, Inc., Austin, TX, United States

**Keywords:** FMR1 premutation, carrier screening, AGG interruptions, genetic counseling, risk assessment, full mutation expansion

## Abstract

**Introduction:** Fragile X syndrome (FXS) is a common form of X-linked intellectual and developmental disability with a prevalence of 1/4000–5000 in males and 1/6000–8000 in females. Most cases of the syndrome result from expansion of a premutation (55–200 CGGs) to a full mutation (>200 CGGs) repeat located in the 5′ untranslated region of the fragile X mental retardation (*FMR1*) gene. The risk for full mutation expansions increases dramatically with increasing numbers of CGG repeats. Recent studies, however, revealed AGG interruptions within the repeat area function as a “protective factor” decreasing the risk of intergenerational expansion.

**Materials and Methods:** This study was conducted to validate the relevance of AGG analysis for the ethnically diverse Israeli population. To increase the accuracy of our results, we combined results from Israel with those from the New York State Institute for Basic Research in Developmental Disabilities (IBR). To the best of our knowledge this is the largest cohort of different ethnicities to examine risks of unstable transmissions and full mutation expansions among *FMR1* premutation carriers.

**Results:** The combined data included 1471 transmissions of maternal premutation alleles: 369 (25.1%) stable and 1,102 (74.9%) unstable transmissions. Full mutation expansions were identified in 20.6% (303/1471) of transmissions. A total of 97.4% (388/397) of transmissions from alleles with no AGGs were unstable, 79.6% (513/644) in alleles with 1 AGG and 46.7% (201/430) in alleles with 2 or more AGGs. The same trend was seen with full mutation expansions where 40% (159/397) of alleles with no AGGs expanded to a full mutation, 20.2% (130/644) for alleles with 1 AGG and only 3.2% (14/430) in alleles with 2 AGGs or more. None of the alleles with 3 or more AGGs expanded to full mutations.

**Conclusion:** We recommend that risk estimates for *FMR1* premutation carriers be based on AGG interruptions as well as repeat size in Israel and worldwide.

## Introduction

Fragile syndrome (FXS; OMIM 300624) is a common form of X-linked intellectual and developmental disability (Crawford et al., 2001) with a prevalence of 1/4000–5000 in males and 1/6000–8000 in females (de Vries et al., 1997; Crawford et al., 2001; Coffee et al., 2009; Hill et al., 2010). It belongs to a family of more than 40 disorders characterized by repeat instability on transmission from parent to child (Pearson et al., 2005).

Most cases of the syndrome result from expansion of a CGG trinucleotide repeat located in the 5′ untranslated region of the fragile X mental retardation (*FMR1*) gene to more than 200 repeats (full mutation) (Oberle et al., 1991; Verkerk et al., 1991; Yu et al., 1991). The gene encodes the *FMR1* protein (FMRP) (Kremer et al., 1991; Verkerk et al., 1991; Yu et al., 1991), an RNA-binding protein (Ashley et al., 1993) that is essential for normal brain development. The CGG repeat expansion prevents the FMRP expression through hypermethylation of the promoter area. The full mutation allele is silenced in a process resembling X-inactivation (Colak et al., 2014) and resulting in FXS (Hagerman and Hagerman, 2013). This polymorphic CGG repeat has been categorized into four groups according to the CGG repeat length (Maddalena et al., 2001): normal (6–44 repeats), intermediate (45–54 repeats), premutation (55–200 repeats) and full-mutation (>200 repeats). Normal alleles are highly stable on intergenerational transmission while intermediate alleles may expand by a few repeats (Fernandez-Carvajal et al., 2009a). Individuals with premutation alleles are defined as carriers. Many alleles in this range are remarkably unstable and at risk for full mutation expansions even in one generation. As many as 94% of alleles with more than 90 repeats expand to a full mutation (Nolin et al., 2011) and the smallest number of repeats to expand to a full mutation in one generation is 56 (Fernandez-Carvajal et al., 2009a). Expansion to a full mutation occurs almost solely in transmission from mother to child and not from father to daughter although rare exceptions have occurred (Zeesman et al., 2004).

Previous studies have shown that premutation alleles are at risk for full mutation expansions (Fu et al., 1991; Nolin et al., 2003). More recent studies revealed AGG interruptions within the repeat area function as a ”protective factor” decreasing the risk for intergenerational expansion (Nolin et al., 2011, 2013, 2015; Yrigollen et al., 2012).

In Israel, all women who wish to conceive are offered genetic screening for fragile X and other disorders free of charge. Women who carry a fragile X premutation are also offered prenatal diagnosis, either chorionic villous sampling (CVS) or amniocentesis (AC). Alternatively, IVF-PGT-M (*In Vitro* Fertilization with pre-implantation genetic testing for monogenic gene diseases) is offered to couples at high risk (>70 repeats) for expansion to full mutation.

At the present time genetic counseling for *FMR1* premutation carriers in Israel does not include AGG interruptions as a factor in determining risks of intergenerational CGG expansion. We have conducted this study to validate the relevance of AGG analysis for the diverse Israeli population.

## Materials and Methods

### Subjects

Blood samples were collected from women undergoing genetic testing at the Danek Gertner Institute of Human Genetics at Sheba Medical Center, Israel from 2011 to 2017. The study was approved by the IRB committee of Sheba Medical Center. *FMR1* premutation carriers with 55–90 CGG repeats were included in the study because previous studies have shown that the presence of AGG interruptions does not reduce risk of a full mutation expansion in females with >90 CGG repeats (Nolin et al., 2015). To increase the accuracy of our results, we combined results from Israel with those from New York State Institute for Basic Research in Developmental Disabilities (IBR).

### Molecular Characterization

The number of FMR1 CGG repeats in the polymorphic zone, number of AGG interruptions and position of AGG interruptions were determined using a triplet primed PCR method as reported in previous studies (Lyon et al., 2010; Basehore et al., 2012; Yrigollen et al., 2012; Nolin et al., 2013), using a GeneAmp (PCR system 9700) by Applied Biosystems, AmplideX *FMR1* PCR/CE (Asuragen), and Asuragen kit (Xpansion Interpreter), based on the company’s protocol of AmplideXTM FMR1 PCR reagents (ROU). The FMR1 CGG Primer is specific for CGG repeats and will not hybridize to AGG sequences commonly found in FMR1 alleles. Therefore, signal intensity dips in the CGG RP PCR profile correspond to the presence of interspersed AGG. Based on peak counting and on the haplotype inference of a 5′-bias for AGG, the exact pattern of CGG repeats and AGG interruptions can be inferred in many cases, even in female samples. The accuracy and validity of our systems were assured using AmplideX© PCR process control and mPCR & Sensitivity.

A change in repeat size was defined as a difference of at least one CGG repeat from mother to fetus.

### Statistical Analysis

Statistical analyses were done using ^®^JMP Statistical Discovery software, version 14.0.0 from ^®^SAS Institute Inc., Cary, NC, United States.

Data of each subgroup (Israel and IBR) were analyzed separately to explore possible differences between sets. Obviously, the large difference in sample size makes it difficult to make statistical inference about their similarity. However, in spite of the large difference in sample size both data sets – Israel and IBR show similar properties regarding the combination of maternal CGG repeats and the number of AGG interruptions effect on the probability of transferring a full mutation from women to their siblings. Therefore, it is reasonable to relate to the combined data of both studies as a single set. Logistic regression models were used to determine the effect of the number of CGG repeats and the number of AGG interruptions on the risk of a full mutation expansion. With regard to the number of AGG interruptions and their effect on full mutation expansion a different behavior was identified between women who have 55–70 CGG repeats and women with 70–90 CGG repeats. This binary indicator was used as a parameter in the logistic regression models.

## Results

From 2011 to 2017 a total of 408 FMR1 premutation carriers (55–200 CGG repeats) were identified at the Danek Gertner Institute of Human Genetics at Sheba Medical Center. A total of 362 carriers (89% with 55–90 repeats) were separated into seven categories, each category containing the range of five repeats. Prenatal diagnosis was performed for 430 fetuses (425 pregnancies: 239 males and 191 females) by amniocentesis (37%) or chorionic villus sampling (63%). The expanded allele was transmitted to 198 fetuses (46%).

### Intergenerational Repeat Instability and Risk of Full Mutation Expansions

#### Israel Data

Of the 198 cases where the expanded allele was transmitted, 75 (37.9%) were stably transmitted and 123 (62.1%) increased in size of which 24 (12.1%) were full mutation expansions. The smallest premutation allele to expand to a full mutation had 62 repeats with no AGG interruptions. As reported in previous studies, the frequency of unstable transmissions and full mutation expansions increased with increasing repeat size, reaching nearly 100% when the CGG repeat size exceeded 80 and there were no AGG’s.

#### Combined Results of Israel and Institute for Basic Research (IBR)

**FIGURE 1:**
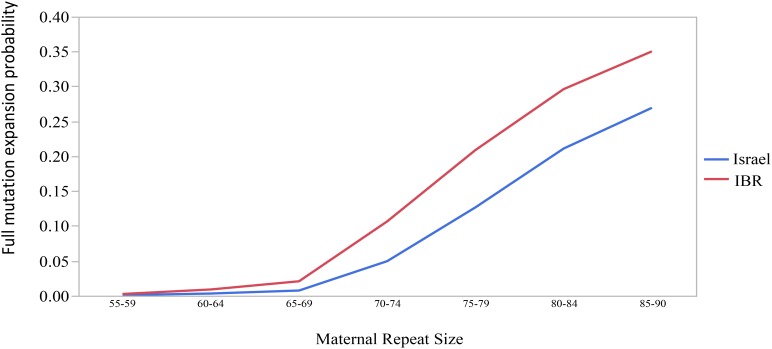
The effect of the maternal number of CGG repeats upon the probability for a full mutation expansion.

**FIGURE 2:**
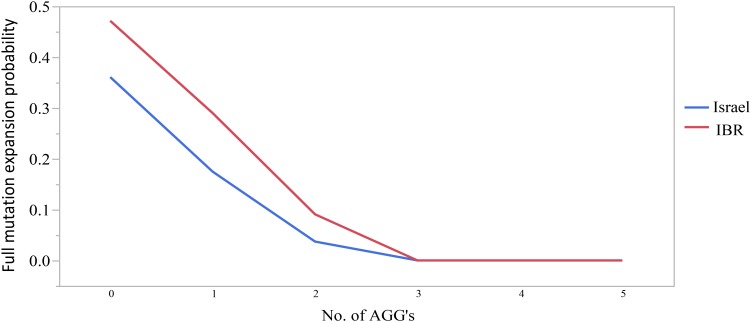
The effect of the number of AGG interruptions upon the probability for a full mutation expansion.

The combined data included 1471 transmissions: 369 (25.1%) stable and 1102 (74.9%) unstable transmissions. A total of 303 full mutation expansions was identified (20.6% of all transmissions). In the IBR dataset the smallest maternal allele to expand to full mutation had 59 repeats. The risk of full mutation expansions by maternal CGG repeats only is presented in Table 1. There was no significant statistical difference in the rate of full mutation expansion between the Israeli and the IBR results. Moreover, in both populations the maternal number of CGG repeats was shown to have a statistically significant effect upon the risk for a full mutation expansion (*p* < 0.001). Figure 1 demonstrates the effect of the maternal number of CGG repeats upon the probability for a full mutation expansion while Figure 2 demonstrates the effect of the maternal number of AGG interruptions upon the probability for a full mutation expansion in both study populations.

**Table 1:** The risk of full mutation (FM) expansion by maternal CGG repeat only.

	Israel		Combined	
Maternal repeat size	No. FM/total		No. FM/total	
size	transmissions	%	transmissions	%
55–59	0/36	0	1/388	0.3
60–64	1/43	2.3	4/300	1.3
65–69	1/48	2.1	7/200	3.5
70–74	3/33	9.1	34/166	20.5
75–79	2/14	14.3	72/153	47.1
80–84	10/13	76.9	94/146	64.4
85–90	7/11	63.6	91/118	77.1
Total	24/198	12.1	303/1471	20.6


### AGG Analysis

#### Israel Data

Logistic regression analysis has shown that AGG interruptions have a significant effect on the risk of a full mutation expansion and the main difference is between those that have some AGG interruptions (1 or 2) and those that do not have them at all (*p* < 0.04) (Table 2). The risk for full mutation expansions was 21.2% for alleles with no AGG interruptions, 9.8% for 1 AGG and 4% in transmissions for alleles with 2 or more AGG interruptions. Table 3 demonstrates the risks for unstable transmissions and full mutation expansions by repeat size and number of AGG interruptions in the Israeli population. Among the carriers with 0–2 AGG interruptions, the AGG interruptions were shown to have a statistically significant “protective” effect against a full mutation expansion (*p* < 0.05). In the sub population of women with a small number of CGG repeats (below 70), full mutations are observed only in those women that do not have AGG interruptions. This result is consistent between the Israeli data and the IBR data.

**Table 2:** The risk of unstable transmission and full mutation expansion according to the number of AGG interruptions among Israeli’s FMR1 premutation carriers with 55–90 CGG repeats.

No. AGG	Stable	Unstable	Full
interruptions	transmissions (%)	transmissions (%)	mutations (%)
0	12.1	87.9	21.2
1	41.5	58.5	9.8
≥ 2	66.0	35.6	4.0


**Table 3:** Unstable transmissions and full mutation expansions sorted by repeat size and number of AGGs among Israeli population.

Maternal
repeat	No.	Total	Unstable		Full
size	AGG’s	transmissions	transmissions	%	mutations	%
55–59	0	6	4	66.7	0	0
	1	9	1	11.1	0	0
	2	15	3	20	0	0
	3	3	0	0	0	0
	4	3	0	0	0	0
60–64	0	10	10	100	1	10
	1	18	8	44.4	0	0
	2	8	1	12.5	0	0
	3	2	1	50	0	0
	4	5	1	20	0	0
65–69	0	23	19	82.6	1	4.4
	1	22	12	54.5	0	0
	2	2	1	50	0	0
	3	1	1	100	0	0
70–74	0	8	7	87.5	1	12.5
	1	18	16	88.9	2	11.1
	2	7	6	85.7	0	0
75–79	0	5	5	100	0	0
	1	7	4	57.1	1	14.3
	2	2	2	100	1	50
80–84	0	8	8	100	7	87.5
	1	4	3	75	2	50
	2	1	1	100	1	100
85–90	0	6	5	83.3	4	66.7
	1	4	4	100	3	75
	2	1	0	0	0	0


#### Combined Data

In the combined data for Israel and IBR, AGG interruptions reduced the risk of unstable transmissions and full mutation expansions. A total of 97.4% of transmissions from alleles with no AGGs was unstable, 79.6% in alleles with 1 AGG and 47.2% in alleles with 2 or more AGGs. The same trend was seen with full mutation expansions where 40% of alleles with no AGGs expanded to a full mutation, 20.2% for alleles with 1 AGG and only 3.2% in alleles with 2 AGGs or more. None of the alleles with 3 or more AGGs expanded to full mutations and 23/27 (85.1%) were transmitted stably. Again, as demonstrated among the Israeli population, the number of AGG interruptions was found to have a statistically significant negative effect upon the risk of a full mutation expansion (*p* < 0.001). Table 4 demonstrates the risks for unstable transmissions and full mutation expansions by repeat size and number of AGG interruptions in the Israeli and IBR populations.

**Table 4:** Unstable transmissions and full mutation expansions sorted by repeat size and number of AGGs based on the combined Israeli and international data.

Maternal
CGG	No.	Total	Unstable		Full	
repeat size	AGG	transmissions	transmissions	%	mutations	%
55–59	0	52	49	94.2	1	1.9
	1	171	91	53.2	0	0
	2	145	21	14.5	0	0
	3	16	0	0	0	0
	4	3	0	0	0	0
	5	1	0	0	0	0
60–64	0	74	73	98.6	4	5.4
	1	121	90	74.4	0	0
	2	96	42	43.8	0	0
	3	7	3	42.9	0	0
	4	2	1	50	0	0
65–69	0	70	66	94.3	7	10
	1	77	63	81.8	0	0
	2	50	34	68	0	0
	3	3	1	33.3	0	0
70–74	0	54	53	98.1	28	51.9
	1	79	77	97.5	6	7.6
	2	33	27	81.8	0	0
75–79	0	60	60	100	43	71.7
	1	65	62	95.4	26	40
	2	28	28	100	3	10.7
80–84	0	51	51	100	45	88.2
	1	66	65	98.5	43	65.2
	2	29	29	100	6	20.7
85–90	0	36	35	97.2	31	86.1
	1	65	65	100	55	84.6
	2	17	16	94.1	5	29.4
Total		1471	1102	74.9	303	20.6


Table 5 summarizes the statistical analysis results of each data set and of the combined data. Since the vast majority of subjects in the different data set had AGG interruptions between zero and two, only those were considered in these models. The odds ratios in Table 5 summarize the change in risk of transferring a full mutation from a woman to its child. The risk of transferring a full mutation increases dramatically when the maternal number of CGG repeats is above 70. The number of AGG interruptions plays an important role in such transfers. When this number is up from zero to one, the risk of transferring a full mutation gets lower and is statistically significant. Looking at the Israeli data, noticeably the change from a single AGG interruption to two interruptions makes a little difference and is not statistically significant. With the fairly small sample size compared to that of the IBR data this result may indicate that the important difference in terms of risk of transferring a full mutation is between those women who have some AGG interruptions and those who do not. In the case of IBR data (and obviously the combined data), all terms in the model are statistically significant though the odds ratios are similar to those obtained for the Israeli data, probably due to the very large sample size.

**Table 5:** Summary of *P*-values, odds ratios and 95% CI of odd ratios from the regression models.

	Parameters	*P*-value	Odds ratio	OR lower 95%	OR upper 95%
Israel data	Repeats size	<0.0001*	26.31	7.24	170.72
	No. of AGG’s (0-1)	0.0374*	0.33	0.11	0.91
	No. of AGG’s (1–2)	0.6503	0.68	0.09	3.22
IBR data	Repeats size	<0.0001*	93.69	49.90	194.42
	No. of AGG’s (0–1)	<0.0001*	0.25	0.16	0.37
	No. of AGG’s (1–2)	<0.0001*	0.14	0.07	0.26
Combined data	Repeats size	<0.0001*	77.48	51.42	122.73
	No. of AGG’s (0–1)	<0.0001*	0.28	0.21	0.36
	No. of AGG’s (1–2)	<0.0001*	0.17	0.11	0.26


*^∗^In the regression models the parameter of CGG repeats (55–69 and 70–90 repeats) was used as a binary indicator.*

## Discussion

To the best of our knowledge this is the largest cohort of different ethnic backgrounds used to examine risks of unstable transmissions and full mutation expansions among *FMR1* premutation carriers. As previously mentioned, the two populations (Israel and IBR) were found to have similar characteristics regarding the effect upon the risk of a full mutation expansion, of both the number of CGG repeats and AGG interruptions. Therefore, the combination of these databases is justified in order to increase the study sample size.

Women who have small premutations with fewer than 70 repeats can have no AGG interruptions to as many as five. Our study suggests that, within this group, only those who have no AGGs are at risk to transmit full mutation alleles. Among women with 70–90 repeats, the risk of transmitting full mutation expansions increases as the number of AGG interruptions decreases.

The female carrier prevalence in Israel is ∼1/150 (Berkenstadt et al., 2007) while in the United States the frequency varies from 1/150 to 1/380 (Cronister et al., 2005; Iong et al., 2011; Seltzer et al., 2012). The global prevalence is 1/250 (Rousseau et al., 1995; Rife et al., 2003; Fernandez-Carvajal et al., 2009b) while the lowest prevalence of 1/788 is found in Korea (Jang et al., 2014).

Previous studies of transmissions of fragile X alleles demonstrated that the risk for full mutation expansions increases dramatically with increasing numbers of CGG repeats (Eichler et al., 1994; Nolin et al., 2003). Those studies provided risk estimates of expansion to the full mutation range depending on the number of CGG repeats and became an important tool for genetic counseling of *FMR1* premutation carriers. However, it has been unclear why some alleles with relatively small repeats expanded to a full mutation while other larger alleles were transmitted stably. Sequence analysis of the expanded alleles in the early 1990’s identified the presence of AGG trinucleotides that are often interspersed at positions 10–11 and 20–21 within the repeat region (Eichler et al., 1994). Nearly 95% of individuals with normal alleles have one or two AGG interruptions whereas unstable alleles contain few or no AGGs (Eichler et al., 1996; Falik-Zaccai et al., 1997). The AGG interruptions may maintain stability on transmission by preventing DNA slippage during replication (Eichler et al., 1994) and engage a “biological brake” that curbs expansion (Jarem et al., 2010). Until recently, however, this hypothesis was difficult to examine due to the lack of technological means that allow reliable and efficient interrogation of AGG structures (Latham et al., 2014).

The introduction of a PCR-based method capable of determining the number and pattern of AGG interruptions (Chen et al., 2010) enabled population studies of AGG interruptions that demonstrated that AGG interruptions have a substantial influence upon the risk of a full mutation expansion in a given number of repeats (Yrigollen et al., 2012, 2014; Nolin et al., 2013, 2015). In order to better estimate the risk of full mutation expansion in the lower range of premutation, the incorporation of AGG analysis has been previously suggested to be included in genetic counseling (Finucane et al., 2012; Monaghan et al., 2013; Biancalana et al., 2015; Committee on Genetics, 2017). Indeed, the importance of AGG analysis in identifying the specific alleles at the highest risk for a full mutation expansion has been accumulating and may assist in the decision making whether undergo invasive fragile X prenatal testing (Nolin et al., 2015). In Israel and some other countries, however, AGG analysis has not become a part of the standard genetic counseling due to lack of validation in the local population.

In Israel, genetic screening for fragile X is recommended and free of charge for every woman in her reproductive years. Genetic counseling currently provided to *FMR1* premutation carriers in Israel is based solely on the number of CGG repeats with the risk of a full mutation expansion calculated from published data collected from the Israeli population. Our study demonstrates that the effect of AGG interruptions upon the risk for a full mutation expansion is relevant to the Israeli population as well. We have shown a significant increase in the overall risk for a full mutation for alleles with no AGGs compared to alleles with 1 or more AGG interruptions (*p* < 0.05). However, full expansion may still happen even in cases with 2 AGG interruptions particularly in whom whole CGG repeat is long. We refer to the difference in the *p*-value between the Israel and IBR groups to be related to the large difference in the sample size as the statistically significant effects were found for the two variables (CGG, AGG) between the two populations.

**FIGURE 3:**
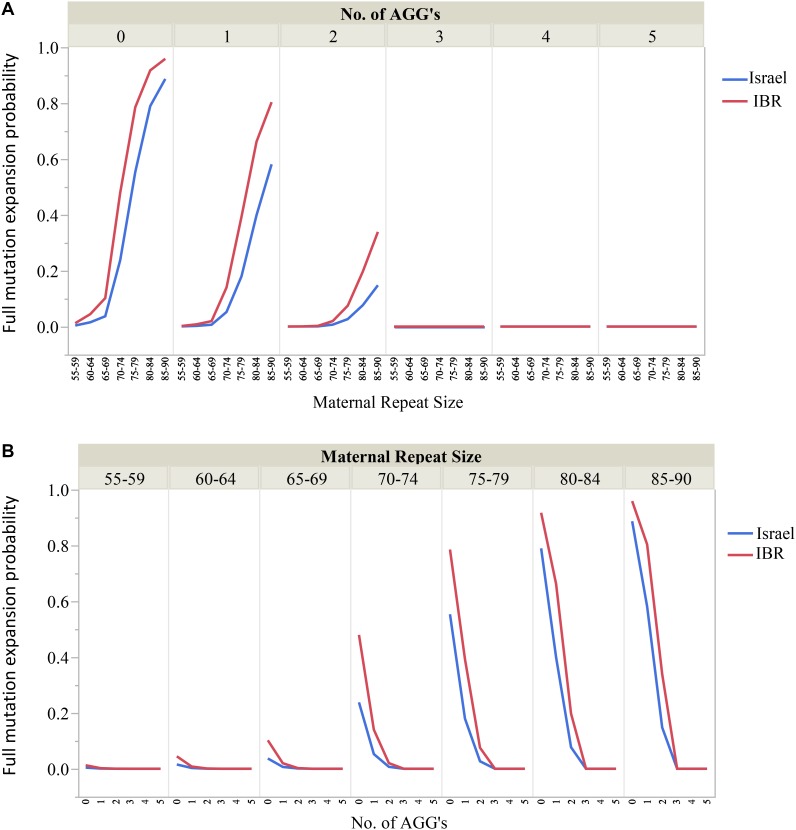
**(A,B)** The effect of the maternal number of CGG repeats and AGG interruptions upon the risk for a full mutation expansion.

Moreover, the risks for unstable transmissions and full mutation expansions in this study differ significantly from the older data currently in use. The AGG interruptions, a relatively “new” variable has proven to be a substantial and significant factor for risk estimates for full mutation expansions among carriers of different ethnicities. For example, our data suggest that for FMR1 premutation carriers with 55–64 CGG repeats and 2 AGG interruptions there is no apparent risk for full mutation expansions. Carriers with 70–74 CGG repeats should be aware of the differences in expansion risk for alleles with 2 AGGs compared to those with none. Figures 3A,B demonstrate graphically the considerable effect that AGG interruptions have upon the risk for a full mutation expansion, which can no longer be overlooked. Further, large scale studies are needed to confirm these recommendations. However, our study demonstrates that a consideration of AGG interruptions should become an integral part of genetic counseling for *FMR1* premutation carriers in Israel and worldwide.

## Ethics Statement

This study was carried out in accordance with the recommendations of Guidelines for Clinical Trials in Human Subjects, IRB/Ethics (Helsinki) Committee of Sheba Medical Center. The protocol for this retrospective analysis of clinical data was approved by the IRB/Ethics (Helsinki) Committee of Sheba Medical Center. The approval specifically waived the need for written informed patient consent.

## Author Contributions

ND edited the manuscript and collected the data. LR-L, LM-H, and MB collected the data. EP, AG, NT, GL, and AH edited the manuscript. YC, SN, and SE initiated and edited the manuscript.

## Conflict of Interest Statement

AH and GL are full-time employees of and have stock options in Asuragen, Inc.The remaining authors declare that the research was conducted in the absence of any commercial or financial relationships that could be construed as a potential conflict of interest.
